# Metabolomic Differences between Viable but Nonculturable and Recovered *Lacticaseibacillus paracasei* Zhang

**DOI:** 10.3390/foods12183472

**Published:** 2023-09-18

**Authors:** Huiying Wang, Yuhong Zhang, Lixia Dai, Xiaoyu Bo, Xiangyun Liu, Xin Zhao, Jie Yu, Lai-Yu Kwok, Qiuhua Bao

**Affiliations:** 1 Key Laboratory of Dairy Biotechnology and Engineering, Ministry of Education, Inner Mongolia Agricultural University, Hohhot 010018, China; m15049697861@163.com (H.W.); z1305404736@163.com (Y.Z.); dailixia2020@163.com (L.D.); bxy1610@163.com (X.B.); 15847755200@163.com (X.L.); babyzhaox@163.com (X.Z.); yujie8301@sina.com (J.Y.); kwok_ly@yahoo.com (L.-Y.K.); 2 Key Laboratory of Dairy Products Processing, Ministry of Agriculture and Rural Affairs, Inner Mongolia Agricultural University, Hohhot 010018, China; 3 Inner Mongolia Key Laboratory of Dairy Biotechnology and Engineering, Inner Mongolia Agricultural University, Hohhot 010018, China

**Keywords:** probiotic, lactobacilli, viable but nonculturable state (VBNC), cell sorting, cell recovery

## Abstract

The fermentation process can be affected when the starter culture enters the viable but nonculturable (VBNC) state. Therefore, it is of interest to investigate how VBNC cells change physiologically. *Lacticaseibacillus* (*L*.) *paracasei* Zhang is both a probiotic and a starter strain. This study aimed to investigate the metabolomic differences between VBNC and recovered *L*. *paracasei* Zhang cells. First, *L*. *paracasei* Zhang was induced to enter the VBNC state by keeping the cells in a liquid de Man–Rogosa–Sharpe (MRS) medium at 4 °C for 220 days. Flow cytometry was used to sort the induced VBNC cells, and three different types of culture media (MRS medium, skim milk with 1% yeast extract, and skim milk) were used for cell resuscitation. Cell growth responses in the three types of recovery media suggested that the liquid MRS medium was the most effective in reversing the VBNC state in *L. paracasei* Zhang. Metabolomics analysis revealed 25 differential metabolites from five main metabolite classes (amino acid, carbohydrate, lipid, vitamin, and purine and pyrimidine). The levels of L-cysteine, L-alanine, L-lysine, and L-arginine notably increased in the revived cells, while cellulose, alginose, and guanine significantly decreased. This study confirmed that VBNC cells had an altered physiology.

## 1. Introduction

The production of food frequently uses lactic acid bacteria (LAB), a class of Gram-positive bacteria that ferment carbohydrates [1]. During industrial food production processes, some LAB may be exposed to unfavorable growth conditions, such as extreme temperature, nutrient restriction, and low pH [2]. Some bacteria enter the viable but nonculturable (VBNC), which helps cells withstand a harsh environment and external stress. Cells in this state can endure for a very long time while only having very low metabolic activity [3]. VBNC cells, on the other hand, may recover by resuming normal activity and becoming cultivable again if the external stress is removed or the unfavorable environment is changed [4]. Most research on the physiology and metabolism of the VBNC state has been on Gram-negative bacteria, many of which are pathogens. There is currently a lack of knowledge on Gram-positive bacteria in this aspect, especially probiotics [5].

The concept of VBNC was first proposed by Xu et al. (1982), who described the presence of VBNC in river and marine environments, including *Escherichia coli* and *Vibrio cholerae* [6]. It has been confirmed that more than 100 microorganisms (including bacteria and fungi) can enter the VBNC state [7]. Various methods, such as raising environmental temperature and adding organic matter or other essential nutrient elements, have been used to revive microorganisms in the VBNC state [8,9]. However, the strategy to recover from the VBNC state is microbe-specific, requiring extensive experimental testing. Food safety and human health may be at risk if pathogenic bacteria shift from the VBNC state [10]. The recovery of nonpathogenic or even probiotic bacteria, on the other hand, may make it simpler to explore inaccessible microbial resources in nature [7]. Regardless of whether the objective is to prevent the reversal of pathogenic microbes in the VBNC state or to recover beneficial ones, our understanding of the physiology and metabolism of VBNC bacterial cells is still rather limited.

Flow cytometry is a crucial nonculture technique for detecting cell activity during the induction and recovery of VBNC cells [11]. This method rapidly moves a focused beam through fluid streams to analyze cells, measuring forward scatter and side scatter at specific wavelengths, detecting bacterial activity, and sorting cells based on the obtained information [12]. Cell activity has been widely detected using fluorescent labeling technology [13]. Based on cell viability and activity, cell subpopulations can be differentiated using flow cytometry and fluorescent labeling (SYTO 9 and propidium iodide [PI]) [14]. SYTO 9 dye can penetrate any type of cell, whereas PI can only bind to DNA through damaged cell membranes [15,16]. Therefore, VBNC cell subpopulations can be effectively chosen and collected for further experimentation using a cell sorter like the MoFlo Astrios^EQ^ based on cell viability and activity staining patterns [17].

The processes of entering and exiting the VBNC state are intricate and may involve significant alterations in the physicochemical and metabolic state of the cells [18]. However, the mechanism of microbial VBNC state recovery is still unclear, especially from the metabolomic perspective. Metabolomics has the potential to give a more accurate snapshot of the actual physiological state of the cell by examining the full range of the metabolites present in a microorganism and tracking the overall results of the interactions between its development processes and the environment [19]. Changes in gene and protein expression are reflected at the metabolomics level [20]. A commonly employed analytical technique in metabolomics research is ultra-performance liquid chromatography quadrupole time-of-flight tandem mass spectrometry (UPLC-Q-TOF-MS/MS), which has the advantages of high peak resolution, quick analysis time, and high detection sensitivity [21,22]. It is suitable for separating and analyzing traces of components in various complex samples [23]. Since there is little information on the physiology, metabolism, and recovery of VBNC bacteria, particularly in probiotics, it would be of interest to use the power of metabolomics analysis to understand the biology of VBNC bacteria and identify the key metabolites and metabolic pathways that are involved in switching between the VBNC and normal cell states.

*Lacticaseibacillus paracasei* Zhang (*L. paracasei* Zhang) is a strain of lactic acid bacteria with good probiotic properties. It was isolated from a koumiss sample collected in Inner Mongolia, China [24]. This probiotic strain shows strong resistance to acid and bile salt stress [25]. It has been shown to have a variety of probiotic effects in both animal and human intervention trials, including lowering blood lipids, regulating the immune system, reducing oxidative stress, and inhibiting gut pathogens [26,27]. This bacterium has been widely used in food, fermentation, medicine, and other fields [28,29]. Our earlier research demonstrated that when *L. paracasei* Zhang cells were kept in a cold de Man–Rogosa–Sharpe (MRS) medium for an extended period, the cells could be induced to enter the VBNC state [30]. However, no study has yet examined the recovery of VBNC *L. paracasei* Zhang.

This study used a metabolomics approach to identify the optimal conditions for reviving *L. paracasei* Zhang from the VBNC state and the ensuing physiological changes. The *L. paracasei* Zhang strain was induced into the VBNC state, and VBNC cells were collected by a cell sorter and grown in three different recovery media to identify the optimal resuscitation strategy. The metabolomic differences between the VBNC and recovered cells were then examined using UPLC-Q-TOF-MS/MS.

## 2. Materials and Methods

### 2.1. Bacterial Strain and MRS Medium Formulation

*Lacticaseibacillus paracasei* Zhang was preserved and provided by the Key Laboratory of Dairy Biotechnology and Engineering, Ministry of Education, Inner Mongolia Agricultural University. The strain was deposited in the China General Microbiological Culture Collection Center (deposit number: 1697). Each liter of the liquid MRS medium used in this study comprised: peptone from soybean (10 g), beef extract (10 g), yeast extract (5 g), glucose (20 g), Tween-80 (1 g), potassium phosphate dibasic (2 g), sodium acetate anhydrous (5 g), trisodium citrate dihydrate (2 g), magnesium sulfate (0.2 g), manganese sulfate monohydrate (54 mg). The medium was adjusted to pH 6.5 and autoclaved at 121 °C for 15 min.

### 2.2. Cell Activation and Induction of the VBNC State

*Lacticaseibacillus paracasei* Zhang was stored in lyophilized tubes. To activate the bacteria, the freeze-dried cells were inoculated in 5 mL of MRS medium (Oxoid, Basingstoke, UK), and the inoculated culture was incubated at 37 °C for 24 h. The procedure was repeated three times. *Lacticaseibacillus paracasei* Zhang was induced to enter the VBNC state by keeping the activated cells in the liquid MRS medium (pH 3.8) at 4 °C for about 220 days [30].

### 2.3. Sorting VBNC L. paracasei Zhang Cells by Flow Cytometry

Flow cytometry in combination with fluorescent staining (SYTO 9 and PI; purchased from Dojindo Laboratories, Shanghai, China) was used to differentiate VBNC cells from dead or noninduced cells. Briefly, four clean centrifuge tubes (2 mL) were labeled as the negative control, PI-positive, SYTO 9-positive, and PI-SYTO 9-double-positive. Samples of *L. paracasei* Zhang VBNC cells (5 mL) were centrifuged at 4000× *g* for 5 min. Pelleted cells were washed twice and adjusted to a cell density of about 10^6^ CFU/mL with phosphate-buffered saline. Then, 1 mL of the diluted cells was added to each of the four labeled centrifuge tubes. For the negative control tube, only the bacterial suspension was added. For the other three centrifuge tubes, appropriate reagents were added: 10 μL of PI stain (0.1 mM/L) in PI-positive; 10 μL of SYTO 9 stain (0.2 mM/L) in SYTO 9-positive; 10 μL each of SYTO 9 and PI stains at the respective aforementioned concentrations in PI-SYTO 9-double-positive. The three cell preparations were gently mixed for 30 s and incubated for 15 min at room temperature in the dark before flow cytometry analysis and cell sorting on a MoFlo Astrios^EQ^ flow cytometer (Beckman Coulter, Inc., Indianapolis, IN, USA). Cells that entered the VBNC state were processed in the sorting mode and collected in designated sterile tubes for subsequent recovery experiments.

### 2.4. Recovery of VBNC Cells

The results of our extensive literature search found that the MRS medium, 10% skim milk + 0.1% yeast powder, and skim milk were mostly chosen for the reactivation of lactobacilli [31]. Therefore, the collected VBNC cells were inoculated (2% inoculum) into 5 mL of liquid MRS medium, skim milk with 1% yeast extract, and skim milk, respectively, and incubated at 37 °C for 24 h. The cells were subcultured for three rounds in the respective growth media as described above. Then, 0.5 mL of the resuscitated bacterial suspension of each culture was serially diluted ten-fold in 0.85% NaCl solution and subjected to pour plate count on MRS agar to determine the optimal growth medium for cell recovery. The inoculated agar plates were incubated aerobically at 37 °C for 48 h before colony counting.

### 2.5. Assessing the Viability and Activity of Recovered VBNC Cells

The cellular activities of the recovered VBNC cells were assayed using a LIVE/DEAD BacLight Bacterial Viability Lit L7012 (Thermo Fisher Scientific Inc., Waltham, MA, USA). The staining kit functions via two fluorescent stains, SYTO 9 and PI. Briefly, 500 μL of cell suspensions of recovered bacteria were centrifuged at 4000× *g* for 5 min, washed twice with, and resuspended (500 μL) in phosphate-buffered saline. Then, a 1.5 μL dye mix (1.5 μL each of SYTO 9 and PI dissolved in 100 μL of dimethyl sulfoxide) was added. The cell mixtures were gently mixed and incubated in the dark for 15 min before observing their cellular activity using a fluorescence microscope (Leica Microsystems, Wetzlar, Germany). Viable and active cells having an intact plasma membrane appeared green, while dead bacteria with a damaged cell membrane appeared red [32].

### 2.6. UPLC-Q-TOF-MS/MS Analysis

***Sample pre-treatment for metabolomics analysis.*** Samples of VBNC and resuscitated *L. paracasei* Zhang cells were prepared according to the method described previously [33]. Briefly, each sample (1 mL) was transferred to a 5 mL centrifuge tube and mixed thoroughly with 1/3 volume of acetonitrile. The mixture was centrifuged at 10,000× *g* for 10 min. The supernatant was aspirated and transferred to another new 5 mL centrifuge tube containing 1/3 volume of acetonitrile. The mixture was left at 4 °C for 2 h before centrifugation at 12,000× *g* for 5 min. The supernatant was then concentrated by rotary evaporation for 9 h, re-dissolved by adding 1 mL of 40% acetonitrile solution and centrifuged at 10,000× *g* for 5 min. Finally, the supernatant was filtered through a 0.22 µm microporous membrane. A quality control sample was prepared by mixing an equal volume (15 µL) of all samples, which was applied between every six samples to evaluate the instrumental stability during the sample runs and data collection process.

***UPLC-Q-TOF-MS/MS analysis.*** Metabolomics analysis was performed on the prepared samples using UPLC-Q-TOF-MS/MS (Waters Corporation, Milford, MA, USA). The pretreated samples were separated using a reversed-phase system with a BEH C18 column (1.7 µm, 2.1 mm × 100 mm). The sample injection volume was 10 µL. The flow rate was 0.45 mL/min. The column chamber temperature was 35 °C. Conditions in positive ion mode: mobile phase A, 0.1% formic acid solution; mobile phase B, 0.1% formic acid–acetonitrile solution. Conditions in negative ion mode: mobile phase A, 0.1% ammonia solution; mobile phase B, pure acetonitrile solution. Gradient elution program in positive ion mode: 2 min, 90% A, 10% B; 6 min, 70% A, 30% B; 10 min, 35% A, 65% B; 14 min, 10% A, 90% B; 15 min, 10% A, 90% B; 16 min, 90% A, 10% B. Gradient elution program in negative ion mode: 2 min, 90% A, 10% B; 6 min, 70% A, 30% B; 12 min, 35% A, 65% B; 14 min, 10% A, 90% B; 15 min, 10% A, 90% B; 16 min, 90% A, 10% B. As the rate of gradient change in the elution could affect the peak time and metabolite separation, the rate of gradient change was tested, adjusted, and optimized based on the results of metabolite separation.

Mass spectrometry parameters were selected based on a previous study [34]. Mass spectrometry was conducted using the Q-TOF-MS/MS system (Waters MS Technologies, Manchester, UK) for metabolite detection in both positive and negative ion (ESI^+^/ESI^−^) modes, and the *m*/*z* ratio scan range was between 50 and 1000 *m*/*z*. To ensure the accuracy and reproducibility of the measurements, leucine enkephalin (2 ng/mL) was used as the calibration solution (*m*/*z* ratio set to 556.2771 and 554.2615 *m*/*z* in ESI^+^/ESI^−^, respectively). The specific run parameters were as follows: capillary voltage, 2.5 kV; sample cone well voltage, 40 kV; ion source temperature, 100 °C; desolvent gas temperature, 350 °C; desolvent gas flow rate, 600 L/h; and cone well gas flow rate, 50 h/L.

***Data processing and multivariate statistical analysis.*** The original metabolic data obtained by UPLC-Q-TOF-MS/MS were imported into the Progenesis QI 2.2 (Waters Corporation, Milford, DE, USA) software for data processing. The transformed and preprocessed data were imported into the SIMCA 14.1 software for multivariate statistical analysis and visualized by Metaboanalyst 5.0. Differential metabolites were identified based on a threshold of *p* < 0.05, variable importance in projection (VIP) ≥ 1, and fold change (FC) ≥ 2 between two compared sample groups. Based on the m/z ratio, retention time, and fragment information, significantly different metabolites were identified. Then, Kyoto Encyclopedia of Genes and Genomes pathway analysis was performed using the *Bacillus subtilis* pathway database as a reference to identify differentially abundant metabolic pathways.

## 3. Results and Discussion

### 3.1. Sorting L. paracasei Zhang VBNC Cells by Flow Cytometry

**Figure 1:**
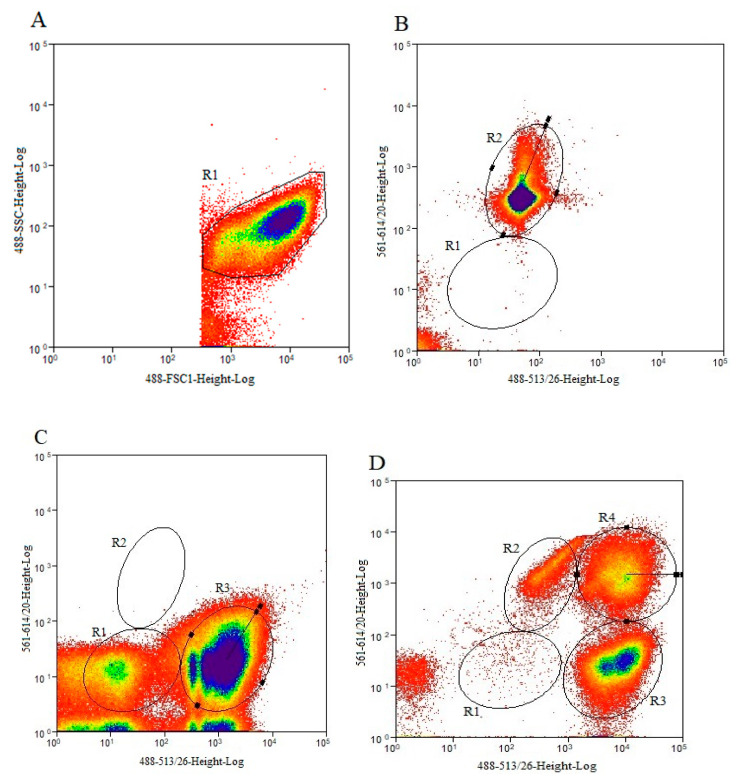
Flow cytometry combined with fluorescence labeling to differentiate live, dead, and viable but nonculturable (VBNC) *Lacticaseibacillus paracasei* Zhang. Dot plots of cells of (**A**) side scatter (SSC) against forward scatter (FSC); (**B**) propidium iodide (PI) staining; (**C**) SYTO 9 staining; (**D**) SYTO 9/PI double staining. R1 in (**A**), R2 in (**B**), R3 in (**C**), and R4 in (**D**) indicate bacteria at all physiological states, dead, live, and VBNC cells, respectively.

The results of cell sorting are shown in Figure 1. As *L. paracasei* Zhang transitioned into the VBNC state, the permeability of the cell membrane was changed. The ability of the SYTO 9 dye to penetrate any cell and PI to only penetrate damaged cell membranes allowed for the differentiation between active and dead cells when SYTO 9 and PI were combined. The staining pattern enabled the partitioning and sorting of VBNC cells.

Cell sorting was based on the staining pattern, which reflected the physiological state of the cells. The negative control consisted of cells that had not been stained; the PI-positive and SYTO 9-positive controls consisted of cells that had been stained by PI and SYTO 9, respectively. The double-positive cells were VBNC cells which were sorted and collected (Figure 1A–D). Figure 1A shows a dot plot of forward scatter (FSC) and side scatter (SSC); cells in the R1 region represent bacterial cells of all physiological states. Figure 1B,C were the positive controls for PI and SYTO 9, respectively; thus, the R2 region in Figure 1B and R3 region in Figure 1C represent the dead and live bacterial cells, respectively. Figure 1D shows a dot plot of SYTO 9/PI-double-staining cells, in which cells were partitioned into three groups: R2 (dead cells), R3 (live cells), and R4 (VBNC cells). Cells in the VBNC state were sorted and collected accordingly.

### 3.2. Resuscitation of VBNC Cells

The sorted VBNC *L. paracasei* Zhang cells were resuscitated in three different types of recovery growth media, namely skim milk, skim milk with 1% yeast extract, and liquid MRS medium. The number of viable cells in the three different recovery media was enumerated by pour plate count, revealing a substantially higher viable count in the culture resuscitated in the liquid MRS medium (8.85 × 10^8^ CFU/mL) than in the skim milk (3.87 × 10^7^ CFU/mL) and skim milk with 1% yeast extract (7.32 × 10^7^ CFU/mL). Therefore, the most effective recovery medium for reversing the VBNC state in *L. paracasei* Zhang was the liquid MRS medium. One obvious difference between the composition of the MRS medium and the skim milk-based media is the high mineral content of the former. Minerals promote the metabolism and growth of lactobacilli, control osmotic pressure, and enhance enzyme or coenzyme activity [35]. The VBNC state of lactic acid bacteria was previously reported to be reversible by adding nutrients [36]. Given the differences in mineral content between the MRS medium and the two media made of skim milk, it is possible that minerals were the essential components needed to resuscitate the VBNC *L. paracasei* Zhang cells. The purpose of this study, however, was not to specifically identify how each chemical element contributed to the resuscitation of VBNC cells. To confirm our hypothesis, additional research will be required.

### 3.3. Growth Curves of Normal and Recovered L. paracasei Zhang

**Figure 2:**
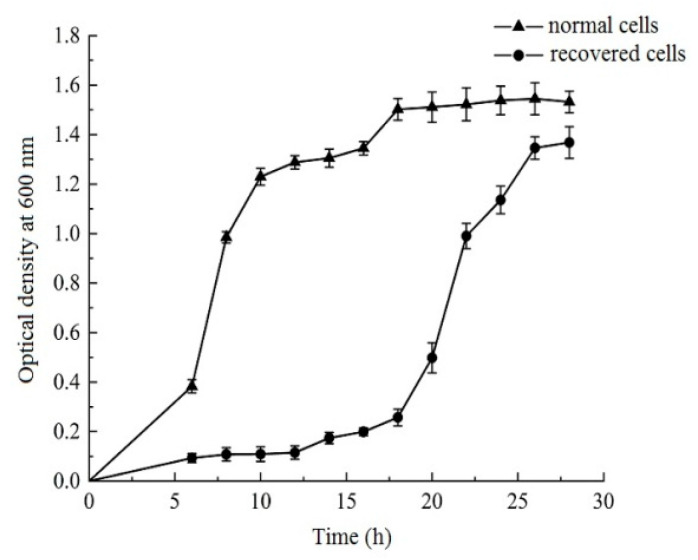
The growth curves of normal and recovered viable but nonculturable *Lacticaseibacillus paracasei* Zhang. Error bars represent SD.

To demonstrate that VBNC *L. paracasei* Zhang were successfully recovered, we compared the growth of normal and recovered *L. paracasei* Zhang in liquid MRS medium (Figure 2). Both the normal and recovered cultures exhibited typical sigmoidal growth curves. However, the normal culture grew more quickly than the recovered culture, with a much shorter lag phase and an earlier logarithmic phase than the recovered culture. After 18 h of growth, when the normal culture had already reached the stationary phase, the recovered culture only reached the logarithmic phase. Furthermore, the optical density of the normal culture (at 600 nm) was still clearly higher than that of the recovered culture at the end of the monitored period (28 h). These findings suggest that, despite the recovered culture regaining the ability to engage in active growth, its growth was still constrained when compared with the normal culture.

### 3.4. Evaluation of the Activity of Recovered L. paracasei Zhang by Fluorescent Microscopy

**Figure 3:**
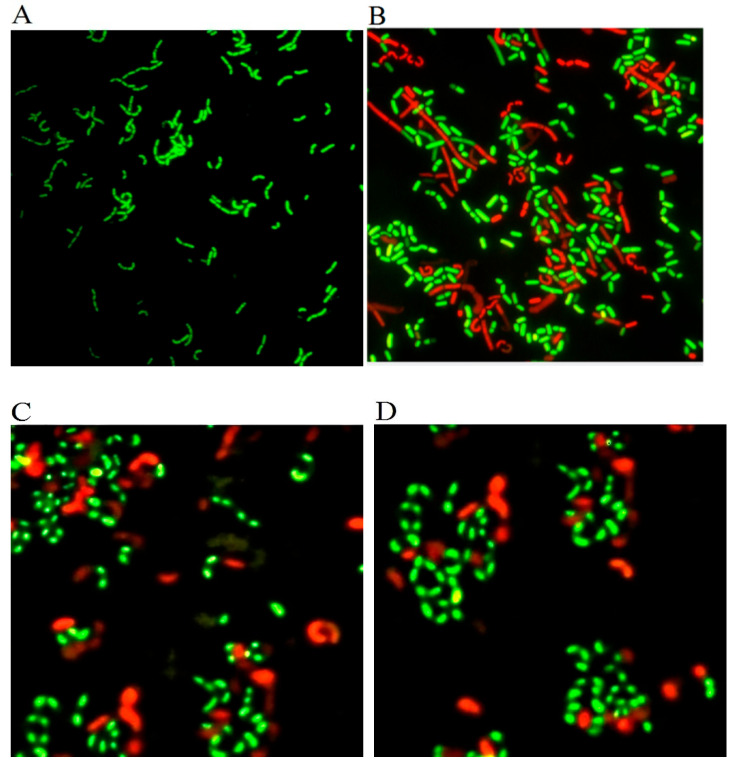
Fluorescent micrographs of cell activity and viability staining by the LIVE/DEAD BacLight^TM^ assay kit. *Lacticaseibacillus paracasei* Zhang in (**A**) normal state, recovered in (**B**) liquid de Man–Rogosa–Sharpe medium, (**C**) skim milk with 1% yeast extract, and (**D**) skim milk; magnification ×1000.

To examine the activity of VBNC cells recovered after three rounds of subculture in liquid MRS medium, skim milk with 1% yeast extract, and skim milk, they were subjected to staining by the LIVE/DEAD BacLight^TM^ kit and fluorescence microscope (Figure 3). The morphology of the cells recovered in the liquid MRS medium resembled that of normal cells, appearing as straight rods with a dispersed cell arrangement. In all three recovered cell cultures, there were more green (active/live) cells than red (inactive/dead) cells, but the fluorescent staining intensity, size, and morphology of recovered cells in the liquid MRS medium resembled normal cells more than those revived in the two skim milk-based media. These results suggested that the culture revived in the MRS medium had a higher activity level than the other two recovered cultures, which is consistent with cell growth behavior. The variation in the level of VBNC cell recovery using various culture media is consistent with a prior report [37].

### 3.5. Metabolomic Differences between VBNC and Resuscitated Cells

**Figure 4:**
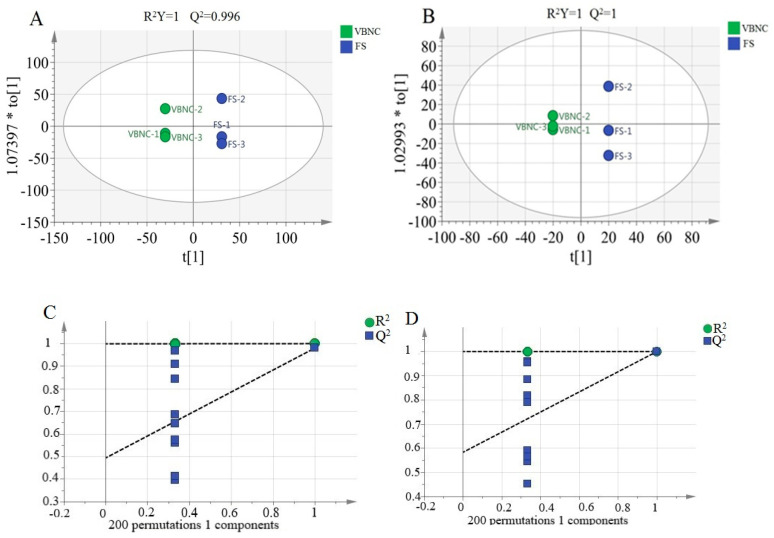
Orthogonal projections to latent structures discriminant analysis (OPLS-DA) of metabolomics data of viable but nonculturable (VBNC) and recovered *Lacticaseibacillus paracasei* Zhang (FS). OPLS-DA plots and results of permutation tests (200 random permutations) of metabolomics data generated in the (**A**,**C**) positive and (**B**,**D**) negative ion modes, respectively. In (**A**,**B**), the subfix letter of the sample code represents the specific replicate sample. The two indicators, R^2^Y and Q^2^, represent the model interpretation rate and predictive ability, respectively. In (**C**,**D**), R^2^ and Q^2^ from 200 permutation tests in the OPLS-DA model are plotted. The *y*-axis shows R^2^ and Q^2^, whereas the *x*-axis shows the correlation coefficient of permuted and observed data. The two points on the right represent the observed R^2^ and Q^2^. The cluster of points on the left represents 200 permuted R^2^ and Q^2^. Dashed lines denote corresponding fitted regression lines for observed and permutated R^2^ and Q^2^.

Metabolomics analysis was performed on VBNC cells and MRS-medium-recovered cells using UPLC-Q-TOF-MS/MS analysis. The metabolomics dataset comprised 1524 and 2149 metabolites in the ESI^+^ and ESI^−^ modes, respectively. The metabolomics data of cells in the two physiological states were then compared using orthogonal projections to latent structures discriminant analyses (OPLS-DA). In OPLS-DA, the two indicators, R^2^Y and Q^2^, represent the model interpretation rate and predictive ability, respectively. The R^2^Y and Q^2^ values of the OPLS-DA models of data obtained in both ESI^+^ and ESI^-^ modes were very high, 1 or very close to 1 (ESI^+^ mode: R^2^Y = 1, Q^2^ = 0.996; ESI^−^ mode: R^2^Y = 1, Q^2^ = 1; Figure 4A,B), suggesting a high interpretation rate and predictive ability in both models.

A permutation test is necessary to confirm whether OPLS-DA results are false positives because the OPLS-DA analysis method is susceptible to overfitting in the case of a small sample size of metabolic data [38]. The OPLS-DA models created from the data of the positive and negative ion modes were therefore evaluated with permutation tests (200 random permutations), and the intercept between the regression line and the *Y*-axis obtained from the permutation tests was used as a standard to determine whether the models were overfitted. The intercepts of R^2^ and Q^2^ in both models were smaller than the explanatory and predictive values of the model variables (~1 in all cases; Figure 4C,D), suggesting that the models were not overfitted and that the generated models were reliable. The permutation test charts were then used to predict VIP values to identify differential metabolites.

### 3.6. Differential Metabolites between VBNC and Recovered Cells

Twenty-five significantly different metabolites (threshold settings of *p* < 0.05, FC ≥ 2, and VIP ≥ 1) were identified in the complete dataset. The recovered cells had 14 significantly increased and 11 significantly decreased metabolites compared with VBNC cells, and these significantly differential metabolites included amino acids, carbohydrates, lipids, vitamins, purines and pyrimidines, and others (Table 1).

#### 3.6.1. Amino Acids

We found that most identified differential metabolites were amino acids. The recovered cells had higher levels of cysteine, L-glutamic acid, L-arginine, and L-glutamine than the VBNC cells, whereas L-tyrosine showed an opposite trend.

Amino acids are organic compounds that are essential for protein building to support the growth, reproduction, and metabolism of any organism, including lactic acid bacteria [39]. Cysteine is a common sulfur source donor crucial for the metabolism of other sulfur-containing macromolecules, including methionine, thiamine, glutathione, and other common substances in the organism. As an essential building block in protein synthesis, intracellular L-cysteine is essential to maintaining cellular homeostasis [40]. L-glutamic acid is the most abundant free amino acid in the host. Glutamic acid metabolites are essential for bacterial growth because they make up the cell wall peptidoglycan and also play a role in preserving the integrity and homeostasis of cells [41]. In addition, L-glutamine has been reported to effectively reduce oxidative damage in bacteria when under stress and improve the activity of brewer’s yeast [42]. In contrast, less L-tyrosine was detected in the recovered cells than in the VBNC cells. L-tyrosine is one of the three major aromatic amino acids and an important conditionally essential amino acid. Intracellular tyrosine might have been depleted during the resuscitation process as the VBNC cells became more active in cell repair and growth resumption.

There may be a preferential requirement for amino acids in the process of VBNC state reversal for *L. paracasei* Zhang, as suggested by a previous study that found that adding various combinations of amino acids to the recovery growth medium could influence the outcome of recovery from the VBNC state in *E. coli* [43].

#### 3.6.2. Carbohydrates

Four differential carbohydrate metabolites were identified between the VBNC and recovered cells. The recovered cells had significantly more xylooligosaccharide than the VBNC cells, whereas the levels of cellulose, N-acetyl-D-glucosamine1-phosphate, and D-trehalose anhydrous showed opposite trends. Xylooligosaccharide is composed of two to nine xylose molecules linked by β-1,4 glycosidic bonds. In addition to providing energy and promoting the growth of beneficial lactic acid bacteria like bifidobacteria, xylooligosaccharide has several biological functions [44,45]. The accumulation of xylooligosaccharide may be conducive to the recovery of VBNC *L. paracasei* Zhang.

Trehalose is a typical stress metabolite produced in response to external stress or environmental changes, and it is noteworthy that it was one of the three metabolites that were significantly reduced in the recovered cells. As trehalose is associated with harsh external conditions, such as drought, fluctuations in temperature, etc., it could potentially protect cells from external insults [46]. The protective mechanism of trehalose on cells involves enhancing interactions between hydrogen bonds and the lipid bilayer by reconstructing tetrahedral hydrogen-bonded water, increasing the strength of hydrogen bonds between water molecules, and improving cell membrane stability [47]. A decrease in intracellular trehalose concentration may indicate an improved growth environment.

#### 3.6.3. Lipids

The levels of three lipids, including palmitoleic acid, N-anthranilate, and methyl-2-methylvalerate, were lower in the recovered cells compared to VBNC cells. A previous study reported that *L. paracasei* Zhang showed alterations in the cell membrane fatty acid composition by shifting to a higher ratio of unsaturated to saturated fatty acids under the low acid stress of artificial gastric juice [48]. In another study, *L. paracasei* Zhang adaptively evolved after continuous cultivating in a low-acidic MRS medium (adjusted to pH 4.3) for 70 days. Both the ratio of unsaturated to saturated fatty acids (i.e., the degree of unsaturation) and the length of fatty acid chains in the cell membrane lipids changed. These modifications presumably sought to improve the acid tolerance of the bacteria and lessen cell damage brought on by acid stress [49]. Thus, it is not surprising that some differential fatty acids (such as palmitoleic acid [C16:1], an unsaturated fat) decreased in response to the process of cell recovery from the cold and acidic environment.

#### 3.6.4. Other Macromolecules

Some intriguing differential metabolites from other macromolecular classes were found in addition to amino acids, carbohydrates, and lipids. For example, more desulfurized biotin was detected in the cell recovery process. Biotin is a B vitamin that is essential for the growth, development, and metabolism of organisms [50]. Biotin synthase uses the Fe–S cluster to supply sulfur atoms and to produce desulfurized biotin, an essential precursor for biotin synthesis. The increase in the abundance of desulfurized biotin may indicate a more active bacterial growth and metabolism [50].

On the other hand, significantly more guanine, uric acid, and methyl pyrimidine were detected in VBNC *L. paracasei* Zhang than in the recovered cells. Apart from being the major biomolecule for energy storage, purines are the components of the nucleotides that transmit genetic information [51]. Nucleotides are an important class of nitrogenous compounds for maintaining fundamental body functions [52]. Guanine dehydrogenase converts guanine to xanthine, which is further metabolized into uric acid through the action of xanthine oxidase [53]. Possibly, when the bacteria were left in the adverse environment for a prolonged period, they were induced to enter the VBNC state. The cells only maintained a minimal metabolic activity to maximize survival, meanwhile preserving a relatively high level of purines and pyrimidines to await the chances for cellular repair processes to occur. These macromolecules may also have been used to support growth when the cells regained a higher level of biological activity during the recovery process.

### 3.7. Enrichment Analysis of Differential Metabolite Pathways

**Figure 5:**
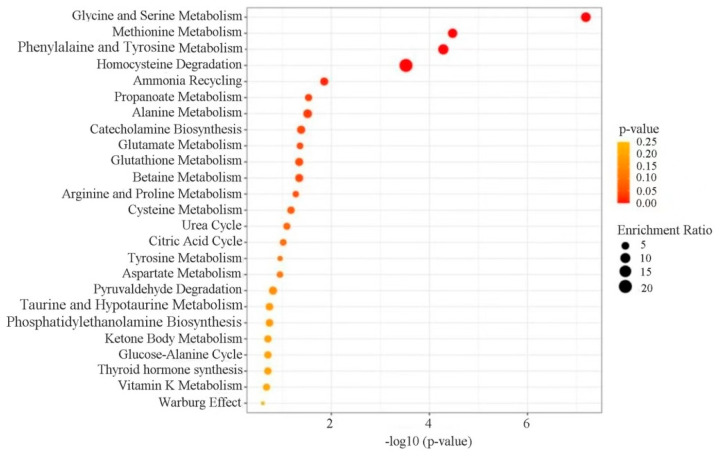
Metabolic pathway enrichment analysis based on the differential metabolites identified between viable but nonculturable (VBNC) and recovered *Lacticaseibacillus paracasei* Zhang cells. The size and color of the circles represent the number of differential metabolites in the specifically enriched pathway and its extent of statistical significance, respectively. A darker circle represents a greater magnitude of significant difference.

Further metabolic pathway enrichment analysis was conducted based on the identified differential metabolites using Metabianalyst 5.0 (Figure 5). A total of 25 significantly enriched metabolic pathways were found, including glycine and serine metabolism, methionine metabolism, phenylalanine and tyrosine metabolism, homocysteine degradation, alanine, ammonia recycling, and others.

Many pathways identified in the pathway enrichment analysis are involved in or are related to amino acid metabolism, suggesting a possible metabolic reshaping from protecting cells against environmental cold and acidic stress for cell maintenance and growth resumption. For instance, glycine is a nonessential amino acid that contributes to the metabolism of essential amino acids in all living things and microorganisms, and it also serves as an important source of energy and nutrition, promoting metabolism along with other classes of macromolecules like carbohydrates and fats [54]. It is also not surprising that the pathway of ammonia recycling was found to be among the top enriched pathways, as it is likely that ammonia was recycled into central amino acid metabolism to maximize nitrogen utilization during the VNBC state recovery process.

The identification of a vast number of differential metabolites and metabolic pathways of amino acid metabolism may also suggest that the activation of these pathways is crucial for VBNC *L. paracasei* Zhang to achieve homeostasis and a physiological balance in the process of transition from an unfavorable to a more favorable environment.

## 4. Conclusions

This study successfully sorted and collected VBNC state *L. paracasei* Zhang cells by flow cytometry. A liquid MRS medium was found to be a more effective growth medium than skim milk-based media for recovering the VBNC cells from cold and acidic stress conditions based on plate counting results. Cell recovery was accompanied by substantial metabolomic changes, including 25 differential metabolites, mainly belonging to amino acids, vitamins, sugars, lipids, and purines and pyrimidines. Fourteen upregulated metabolites (such as L-cysteine, L-alanine, L-lysine, and L-arginine) and eleven downregulated metabolites (such as cellulose, fucoidan, and guanine) were detected in the recovered cells. This study provides interesting information on the recovery of a probiotic bacterium from the VBNC state and the ensuing metabolomic changes during the process.

Cell viability is of particular importance for probiotics, as they need to stay active to exert beneficial effects on the host. Thus, incorporating the right components in the fermentation medium to protect cells from entering the VBNC state during the industrial process would contribute to enhancing the beneficial effects of probiotic-based functional foods or dairy products. Further work will be necessary to pinpoint the exact components responsible for VBNC cell revival and design formulation that would be suitable for use in industrial applications. The current work on investigating the recovery physiological mechanism of lactic acid bacteria in the VBNC state serves as a starting point for such a purpose.

## Figures and Tables

**Table 1:** Differential metabolites between viable but nonculturable state and recovered cells.

Class	Metabolite Identity	Molecular Formula	Retention Time (min)	*m*/*z* Ratio	VIP Value	Log_2_(FC)	*p*-Value
Amino acid	L-Cysteine	C_3_H_7_NO_2_S	0.80	267.07	2.27	3.53	1.12 × 10^−5^
	L-Alanine	C_3_H_7_NO_2_	0.83	134.05	2.23	2.63	3.72 × 10^−6^
	L-Glutamic acid	C_5_H_9_NO_4_	3.65	118.05	2.19	3.03	1.98 × 10^−7^
	L-Lysine	C_6_H_14_N_2_O_2_	15.75	109.77	2.16	6.68	2.70 × 10^−5^
	L-3-Cyanophenylalanine	C_10_H_10_N_2_O_2_	1.09	137.03	2.27	2.21	2.20 × 10^−5^
	L-Glutamine	C_5_H_10_N_2_O_3_	3.64	144.07	2.26	5.84	4.52 × 10^−5^
	L-Arginine	C_6_H_14_N_4_O_2_	14.56	139.10	2.40	5.62	8.97 × 10^−5^
	L-Asparagine	C_9_H_16_N_2_O_5_	3.64	159.08	2.02	3.21	7.26 × 10^−5^
	L-tyrosine	C_9_H_11_NO_3_	3.24	180.07	2.31	−3.14	1.44 × 10^−6^
Carbohydrate	Cellulose	(C_6_H_10_O_5_)n	0.93	295.03	2.10	−2.79	1.02 × 10^−5^
	N-Acetyl-D-glucosamine1-phosphate	C_8_H_16_NO_9_P	0.77	304.06	2.36	−2.58	3.90 × 10^−5^
	Xylooligosaccharide	C_5_H_12_O_5_	1.12	152.06	2.19	3.12	7.70 × 10^−5^
	*D-Trehalose anhydrous*	C_12_H_22_O_11_	2.32	253.64	2.14	−2.87	1.05 × 10^−8^
Lipid	Palmitoleic acid	C_16_H_32_O_2_	5.97	271.23	2.30	−2.23	6.36 × 10^−5^
	N-Anthranilate	C_12_H_16_NO_9_P	0.77	304.06	2.36	−2.58	7.75 × 10^−5^
	Methyl-2-methylvalerate	C_7_H_12_O_4_	0.77	207.04	2.26	−2.86	5.67 × 10^−5^
Vitamin	Desthiobiotin	C_10_H_18_N_2_O_3_	1.11	214.13	2.29	3.12	6.81 × 10^−5^
	Folinic acid	C_20_H_23_N_7_O_7_	5.18	229.09	2.39	−7.60	5.80 × 10^−5^
	Nicotinamide	C_6_H_6_N_2_O	4.52	167.04	2.28	2.31	2.48 × 10^−5^
Purine and pyrimidine	Thymidine	C_10_H_13_N_5_O_5_	3.41	243.08	2.18	2.93	1.05 × 10^−8^
	Guanine	C_5_H_5_N_5_O	4.67	144.07	2.35	−2.53	1.54 × 10^−5^
	2-Methylpyrimidine	C_5_H_6_N_2_	14.56	139.10	2.40	−5.62	7.43 × 10^−6^
Others	3-Oxohexanoyl-CoA	C_27_H_44_N_7_O_18_P_3_S	15.75	938.13	2.34	5.33	3.44 × 10^−5^
	Uric acid	C_5_H_4_N_4_O_3_	0.8	176.05	2.27	3.56	8.95 × 10^−6^
	Hydroxy methylglutaryl coenzyme A	C_27_H_44_N_7_O_20_P_3_S	8.75	950.12	2.03	−5.59	9.77 × 10^−7^

Remarks: FC = fold change, representing the fold difference in metabolite level between the two cell states. A positive value of fold change means a differential increase in the recovered cells compared with the viable but nonculturable state cells.

## Data Availability

The datasets generated and/or analyzed in the course of this study are available from the corresponding author upon reasonable request.

## References

[1] Wang Y.Q., Wu J.T., Lv M.X., Shao Z., Hungwe M., Wang J.J., Bai X.J., Xie J.L., Wang Y.P., Geng W.T. (2021). Metabolism characteristics of lactic acid bacteria and the expanding applications in food industry. Front. Bioeng. Biotechnol..

[2] Hosseini N.M., Hussain M.A., Britz M.L. (2015). Stress responses in probiotic *Lactobacillus casei*. Crit. Rev. Food Sci. Nutr..

[3] Fleischmann S., Robben C., Alter T., Rossmanith P., Mester P. (2021). How to evaluate non-growing cells-current strategies for determining antimicrobial resistance of VBNC bacteria. Antibiotics.

[4] Pan H.X., Ren Q. (2022). Wake Up! Resuscitation of Viable but Nonculturable bacteria: Mechanism and potential application. Foods.

[5] İzgördü Ö.K., Darcan C., Kariptaş E. (2022). Overview of VBNC, a survival strategy for microorganisms. 3 Biotech..

[6] Xu H.S., Roberts N., Singleton F.L., Attwell R.W., Grimes D.J., Colwell R.R. (1982). Survival and viability of nonculturable *Escherichia coli* and *Vibrio cholerae* in the estuarine and marine environment. Microb. Ecol..

[7] Dong K., Pan H.X., Yang D., Rao L., Zhao L., Wang Y.T., Liao X.J. (2020). Induction, detection, formation, and resuscitation of viable but non-culturable state microorganisms. Compr. Rev. Food Sci. Food Saf..

[8] Ayrapetyan M., Williams T., Oliver J.D. (2018). Relationship between the viable but nonculturable state and antibiotic persister cells. J. Bacteriol..

[9] Oliveira M.M., de Almeida F.A.D., Baglinière F., de Oliveira L.L.D., Vanetti M.C.D. (2021). Behavior of *Salmonella Enteritidis* and *Shigella flexneri* during induction and recovery of the viable but nonculturable state. FEMS Microbiol. Lett..

[10] Soto-Beltrá N.M., Lee B.G., Amézquita-López B.A., Quiñones B. (2023). Overview of methodologies for the culturing, recovery and detection of *Campylobacter*. Int. J. Environ. Health Res..

[11] Power A.L., Barber D.G., Groenhof S.R.M., Wagley S., Liu P., Parker D.A., Love J. (2021). The application of imaging Flow Cytometry for characterisation and quantification of bacterial phenotypes. Front. Cell Infect. Microbiol..

[12] McKinnon K.M. (2018). Flow Cytometry: An overview. Curr. Protoc. Immunol..

[13] Wallberg F., Tenev T., Meier P. (2016). Analysis of apoptosis and necroptosis by fluorescence-activated cell sorting. Cold Spring Harb. Protoc..

[14] Bunthof C.J., Bloemen K., Breeuwer P., Rombouts F.M., Abee T. (2001). Flow Cytometric assessment of viability of lactic acid bacteria. Appl. Environ. Microbiol..

[15] Deng Y., Wang L.L., Chen Y.J., Long Y. (2020). Optimization of staining with SYTO 9/propidium iodide: Interplay, kinetics and impact on *Brevibacillus brevis*. Biotechniques.

[16] Pozarowski P., Darzynkiewicz Z. (2004). Analysis of cell cycle by Flow Cytometry. Methods Mol. Biol..

[17] Van Dijk M.A., Gregori G., Hoogveld H.L., Rijkeboer M., Denis M., Malkassian A., Gons H.J. (2010). Optimizing the setup of a Flow Cytometric cell sorter for efficient quantitative sorting of long filamentous cyanobacteria. Cytometry A.

[18] Baffone W., Casaroli A., Citterio B., Pierfelici L., Campana R., Vittoria E., Guaglianone E., Donelli G. (2006). *Campylobacter jejuni* loss of culturability in aqueous microcosms and ability to resuscitate in a mouse model. Int. J. Food Microbiol..

[19] Tang J. (2011). Microbial metabolomics. Curr. Genom..

[20] Chumachenko M.S., Waseem T.V., Fedorovich S.V. (2021). Metabolomics and metabolites in ischemic stroke. Rev. Neurosci..

[21] Wang Y., Liu F., Li P., He C.W., Wang R.B., Su H.X., Wan J.B. (2016). An improved pseudotargeted metabolomics approach using multiple ion monitoring with time-staggered ion lists based on ultra-high performance liquid chromatography/quadrupole time-of-flight mass spectrometry. Anal. Chim. Acta.

[22] Zhao L., Yan F.F., Lu Q., Tang C., Wang X.H., Liu R. (2022). UPLC-Q-TOF-MS and NMR identification of structurally different A-type procyanidins from peanut skin and their inhibitory effect on acrylamide. J. Sci. Food Agric..

[23] Liu F., Wang M., Wang Y., Cao Y.W., Sun Z.L., Chen M.C., Tian X.T., Wan J.B., Huang C.G. (2019). Metabonomics study on the hepatoprotective effect of *Panax notoginseng* leaf saponins using UPLC/Q-TOF-MS analysis. Am. J. Chin. Med..

[24] Ma X.B., Wang L.N., Dai L.X., Kwok L.Y., Bao Q.H. (2023). Rapid detection of the activity of *Lacticaseibacillus casei* Zhang by Flow Cytometry. Foods.

[25] Bai M., Huang T., Guo S., Wang Y., Wang J.C., Kwok L.Y., Dan T., Zhang H.P., Bilige M. (2020). Probiotic *Lactobacillus casei* Zhang improved the properties of stirred yogurt. Food Biosci..

[26] Zhu H., Cao C.J., Wu Z.C., Zhang H.P., Sun Z.H., Wang M., Xu H.Z., Zhao Z., Wang Y.X., Pei G.C. (2021). The probiotic *L. casei* Zhang slows the progression of acute and chronic kidney disease. Cell Metab..

[27] He Q.W., Hou Q., Wang Y., Shen L., Sun Z.H., Zhang H.P., Liong M.T., Kwok L.Y. (2020). Long-term administration of *Lactobacillus casei* Zhang stabilized gut microbiota of adults and reduced gut microbiota age index of older adults. J. Funct. Foods.

[28] Wang J.C., Bai X.Y., Peng C.T., Yu Z.J., Li B.H., Zhang W.Y., Sun Z.H., Zhang H.P. (2020). Fermented milk containing *Lactobacillus casei* Zhang and *Bifidobacterium animalis* ssp. *lactis* V9 alleviated constipation symptoms through regulation of intestinal microbiota, inflammation, and metabolic pathways. J. Dairy Sci..

[29] Wang Y.Y., Yan X., Zhang W.W., Liu Y.Y., Han D.P., Teng K.D., Ma Y.F. (2019). *Lactobacillus casei* Zhang prevents jejunal epithelial damage to early-weaned piglets induced by *Escherichia coli* K88 via regulation of intestinal mucosal integrity, tight junction proteins and immune factor expression. J. Microbiol. Biotechnol..

[30] Bao Q.H., Bo X.Y., Chen L., Ren Y., Wang H.Y., Kwok L.Y., Liu W.J. (2023). Comparative analysis using raman spectroscopy of the cellular constituents of *Lacticaseibacillus paracasei* Zhang in a normal and viable but nonculturable state. Microorganisms.

[31] Hayek S.A., Gyawali R., Aljaloud S.O., Krastanov A., Ibrahim S.A. (2019). Cultivation media for lactic acid bacteria used in dairy products. J. Dairy Res..

[32] Shailaja A., Bruce T.F., Gerard P., Powell R.R., Pettigrew C.A., Kerrigan J.L. (2022). Comparison of cell viability assessment and visualization of *Aspergillus niger* biofilm with two fluorescent probe staining methods. Biofilm.

[33] Sun Y.R., Peng C.T., Wang J.C., Sun H.T., Guo S., Zhang H.P. (2021). Metabolic footprint analysis of volatile metabolites to discriminate between different key time points in the fermentation and storage of starter cultures and probiotic *Lactobacillus casei* Zhang milk. J. Dairy Sci..

[34] Bai J.Q., Guo Q.X., Zhang J., Huang J., Xu W., Gong L., Su H., Luo Y.G., Li J.H., Qiu X.H. (2021). Metabolic profile of dendrobine in rats determined by Ultra-high-performance Liquid Chromatography/Quadrupole Time-of-flight Mass Spectrometry. Comb. Chem. High. Throughput Screen..

[35] Ciosek A., Fulara K., Hrabia O., Satora P., Poreda A. (2020). Chemical composition of sour beer resulting from supplementation the fermentation medium with magnesium and zinc Ions. Biomolecules.

[36] Fera M.T., Maugeri T.L., La Camera E., Lentini V., Favaloro A., Bonanno D., Carbone M. (2008). Induction and resuscitation of viable nonculturable *Arcobacter butzleri* cells. Appl. Environ. Microbiol..

[37] Wei C.J., Zhao X.H. (2018). Induction of viable but nonculturable *Escherichia coli* O157:H7 by low temperature and its resuscitation. Front. Microbiol..

[38] Adebo O.A., Kayitesi E., Tugizimana F., Njobeh P.B. (2019). Differential metabolic signatures in naturally and lactic acid bacteria (LAB) fermented ting (a Southern African food) with different tannin content, as revealed by gas chromatography mass spectrometry (GC-MS)-based metabolomics. Food Res. Int..

[39] Miyajima M. (2020). Amino acids: Key sources for immunometabolites and immunotransmitters. Int. Immunol..

[40] Yin J., Ren W.K., Yang G., Duan J.L., Huang X.G., Fang R.J., Li C.Y., Li T.J., Yin Y.L., Hou Y.Q. (2016). L-Cysteine metabolism and its nutritional implications. Mol. Nutr. Food Res..

[41] Qiao Y.L., Liu G.F., Leng C., Zhang Y.J., Lv X.P., Chen H.Y., Sun J.H., Feng Z. (2018). Metabolic profiles of cysteine, methionine, glutamate, glutamine, arginine, aspartate, asparagine, alanine and glutathione in Streptococcus thermophilus during pH-controlled batch fermentations. Sci. Rep..

[42] Li H., Ma M.L., Luo S., Zhang R.M., Han P., Hu W. (2012). Metabolic responses to ethanol in *Saccharomyces cerevisiae* using a gas chromatography tandem mass spectrometry-based metabolomics approach. Int. J. Biochem. Cell Biol..

[43] Pinto D., Almeida V., Almeida Santos M., Chambel L. (2011). Resuscitation of *Escherichia coli* VBNC cells depends on a variety of environmental or chemical stimuli. J. Appl. Microbiol..

[44] Sun Z.K., Yue Z.H., Liu E., Li X.F., Li C.W. (2022). Assessment of the bifidogenic and antibacterial activities of xylooligosaccharide. Front. Nutr..

[45] Li Z.P., Summanen P.H., Komoriya T., Finegold S.M. (2015). In vitro study of the prebiotic xylooligosaccharide (XOS) on the growth of *Bifidobacterium* spp. and *Lactobacillus* spp.. Int. J. Food Sci. Nutr..

[46] Zhao X., Song X.X., Li Y.P., Yu C.X., Zhao Y., Gong M., Shen X.X., Chen M.J. (2018). Gene expression related to trehalose metabolism and its effect on *Volvariella volvacea* under low temperature stress. Sci. Rep..

[47] Soper A.K., Ricci M.A., Bruni F., Rhys N.H., McLain S.E. (2018). Trehalose in water revisited. J. Phys. Chem. B.

[48] Wu P.Y., An J., Chen L., Zhu Q.Y., Li Y.J., Mei Y.X., Chen Z.M., Liang Y.X. (2020). Differential analysis of stress tolerance and transcriptome of probiotic *Lacticaseibacillus casei* Zhang produced from Solid-State (SSF-SW) and Liquid-State (LSF-MRS) fermentations. Microorganisms.

[49] Wu C.D., Zhang J., Wang M., Du G.C., Chen J. (2012). *Lactobacillus casei* combats acid stress by maintaining cell membrane functionality. J. Ind. Microbiol. Biotechnol..

[50] Marquet A., Bui B.T., Florentin D. (2001). Biosynthesis of biotin and lipoic acid. Vitam. Horm..

[51] Tang Z.W., Ye W.R., Chen H.T., Kuang X.W., Guo J., Xiang M.M., Peng C., Chen X., Liu H. (2019). Role of purines in regulation of metabolic reprogramming. Purinergic Signal..

[52] Maan K., Baghel R., Dhariwal S., Sharma A., Bakhshi R., Rana P. (2023). Metabolomics and transcriptomics based multi-omics integration reveals radiation-induced altered pathway networking and underlying mechanism. NPJ Syst. Biol. Appl..

[53] Cicero A.F.G., Fogacci F., Di Micoli V., Angeloni C., Giovannini M., Borghi C. (2023). Purine metabolism dysfunctions: Experimental methods of detection and diagnostic potential. Int. J. Mol. Sci..

[54] Razak M.A., Begum P.S., Viswanath B., Rajagopal S. (2017). Multifarious beneficial effect of nonessential amino acid, glycine: A review. Oxid. Med. Cell Longev..

